# Four genetic lymphoma-specific events (*MYC*, *BCL2*, *BCL6* and *CCND1*) identified in a high grade B lymphoma case

**DOI:** 10.1038/bcj.2015.99

**Published:** 2015-12-11

**Authors:** A Ittel, C Hélias, M P Wissler, E Toussaint, L Miguet, M P Chenard, L Monier, C Gervais, L Mauvieux

**Affiliations:** 1 Laboratoire d'Hématologie, CHU de Hautepierre, Strasbourg, France; 2 Département de Pathologie, CHU Hautepierre, Strasbourg, France; 3 Département d'Onco-Hématologie, CHU Hautepierre, Strasbourg, France

In the WHO classification,^1^ double or triple-hit lymphoma depicts rare and aggressive lymphomas displaying *BCL2* and/or *MYC* and/or *BCL6* gene rearrangements that are categorized as B-cell lymphomas unclassified, with features intermediate between diffuse B-cell lymphoma and Burkitt lymphoma. Bacher *et al.*^2^ described an interesting series of 10 cases of such neoplasms. In addition, they reported the two first cases displaying four different lymphoma-specific events (quadruple hit) involving the genes *MYC*, *BCL2*, *BCL6* and *CCND1*. We describe here a third case occurring in a 79-year-old male patient suffering from paraesthesias for 4 months who was referred for polyneuritis in a context of poor general condition. Clinical examination showed the presence of numerous axillary, supraclavicular, mediastinal and inguinal lymphadenopathies, neuro-meningeal invasion and skin infiltration. The biopsy of a left arm skin nodule revealed large proliferating cells (Ki-67 80%) stained by anti-CD20, BCL2 and BCL6 antibodies, CD10 and CD23 remaining negative, consistent with the diagnosis of diffuse large B-cell lymphoma (DLBCL), not otherwise specified. Blood cell counts showed 8.1 × 10^9^/l leukocytes, 13.2 g/dl hemoglobin, 166 × 10^9^/l platelets. LDH and β-2 microglobulin were elevated (989 U/I and 9.14 mg/l, respectively). Blood cell film examination showed the presence of 28% abnormal lymphocytes (medium sized, with intense basophilia, irregular nuclear contours, slightly clumped chromatin and frequent prominent nucleoli) suggestive of a high grade lymphoma. Flow cytometry revealed a lambda immunoglobulin light chain restriction. These cells expressed pan-B markers such as CD19, CD20, FMC7, CD22, with weak CD5 and CD43 positivity. CD10 and 23 were negative. Both the morphology and immunophenotype of the blood cells favored a pleomorphic mantle cell lymphoma (MCL) aggressive variant diagnosis. Cytogenetic study performed in the WBCs found a complex hyperdiploid karyotype (47 chromosomes, Figure 1) with a *t*(3;22) translocation involving the *BCL6* and *IGL* genes, a structural abnormality of chromosome 8 resulting in juxtaposition of 5′ *MYC* and *BCL2* in fluorescence *in situ* hybridization (with break of the MYC probe), a derivative chromosome 18 from a *t*(14;18) translocation with fusion of 5′*IGH* and *BCL2*, and a *t*(11;14) complex translocation involving *IGH* and *CCND1* (Figure 2). Other numeral (trisomy 12) and structural abnormalities (involving the 1, 7, 14 and 21 chromosomes) were also detected (Figure 1). Overexpression of cyclin D1 was detected in the WBCs by real-time quantitative PCR, as well as in the skin lesion using immunochemistry. Anti-SOX11 antibody staining was found to be negative. Chemotherapy combining rituximab, ifosfamide, cytosine arabinoside and intrathecal methotrexate was initiated, but the patient died 4 months after the diagnosis. This third case of quadruple-hit lymphoma underlines the complexity of the classification of such aggressive malignancies. Initial rearrangement of the *CCND1* gene characterizes MCL that may harbor in very rare cases additional rearrangements of *MYC* or *BCL6*, but histological transformation to typical large cell lymphoma is not retained in the WHO classification. In addition, cyclin D1 overexpression is considered to be a rare feature in DLBCL. Recently, Ok *et al.*^3^ proposed to reclassify DLBCL with expression of cyclin D1, *CCND1* chromosomal rearrangement and CD5 positivity as an aggressive pleomorphic MCL variant. However, no observation of multiple lymphoma-specific gene rearrangements was described in that study. Juskevicius *et al.*^4^ suggest the existence of a ‘gray zone' in which morphologic, clinical and genetic features are insufficient to segregate lymphomas with overexpression of cyclin D1/translocations involving *CCND1* between blastoid MCL and cyclin D1-positive DLBCL. Regarding the immunophenotyping and molecular data, our case is possibly a genetically unstable aggressive pleomorphic MCL variant, which acquired three additional genetic hits.

## Figures and Tables

**Figure 1:**
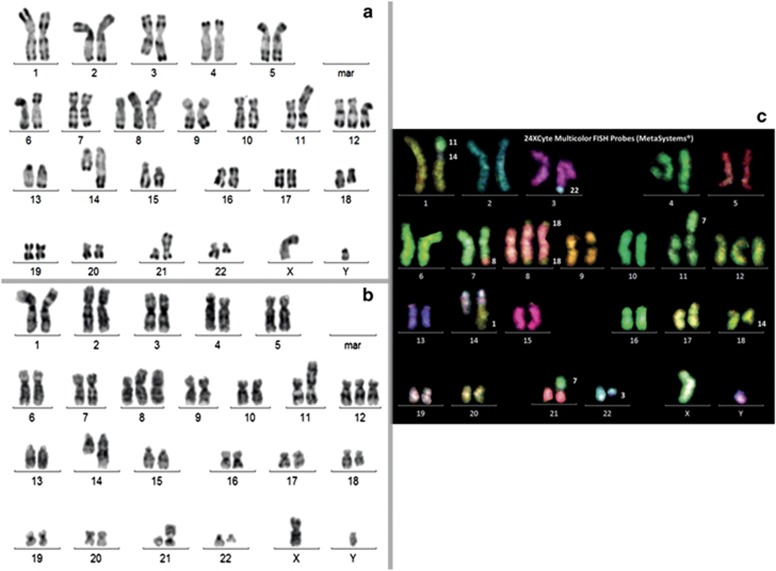
Karyotype in RHG banding (**a**), GTG banding (**b**) and multi-fluorescence *in situ* hybridization (**c**) showing the chromosomes abnormalities: 47,XY,der(1)(11qter->11q13::14q32->14q31::1p21->1qter),*t*(3;22)(q27;q11),der(7)*t*(7;8)(q3?3;q24),der(7;21)(p11;q11),+8,der(8)(18qter->18q21::8?::8p2?1->8q24::18q21->18qter)x2,der(11)*t*(7;11)(q2?1;p15),+12,der(14)*t*(1;14)(p21;q23),der(18)*t*(14;18)(q32;q21).

**Figure 2:**
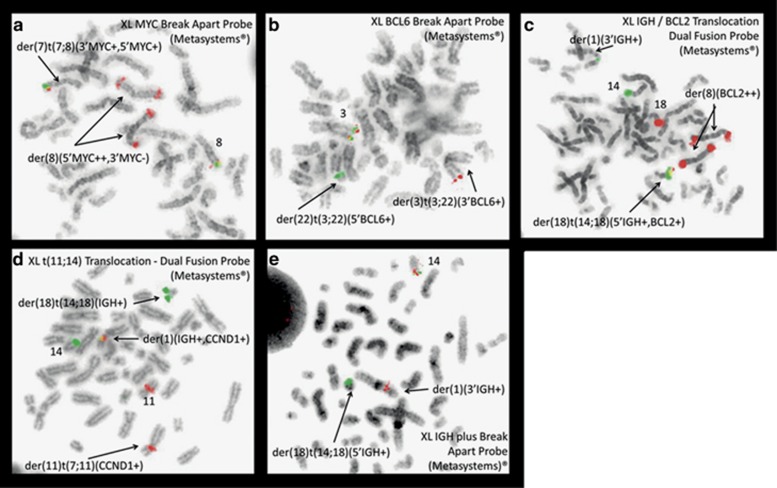
*In situ* hybridization on metaphases showing respectively rearrangements of the *MYC* (**a**), *BCL6* (**b**), *BCL2* (**c**), *CCND1* (**d**) and *IGH* (**e**) genes.
